# Cross-Protection of Inactivated Rabies Vaccines for Veterinary Use against Bat Lyssaviruses Occurring in Europe

**DOI:** 10.3390/v11100936

**Published:** 2019-10-11

**Authors:** Alexandre Servat, Marine Wasniewski, Florence Cliquet

**Affiliations:** French Agency for Food, Environmental and Occupational Health & Safety (ANSES), Nancy Laboratory for Rabies and Wildlife, OIE Reference Laboratory for Rabies, European Union Reference Laboratory for Rabies, European Union Reference Laboratory for Rabies Serology, Technopôle Agricole et Vétérinaire, Domaine de Pixérécourt, CS 40009, 54220 Malzéville, France; marine.wasniewski@anses.fr (M.W.); florence.cliquet@anses.fr (F.C.)

**Keywords:** rabies, veterinary vaccines, cross-protection, bat lyssaviruses

## Abstract

Human rabies vaccines have been shown to induce partial protection against members of phylogroup I bat lyssaviruses. Here, we investigated the capacity of a widely used rabies inactivated vaccine (Rabisin, Boehringer-Ingelheim) for veterinary use to cross-protect mice experimentally infected with European bat lyssavirus 1 (EBLV-1b), European bat lyssavirus 2 (EBLV-2), and Bokeloh bat lyssavirus (BBLV) occurring in Europe. For each lyssavirus, we investigated the efficacy of two different doses of vaccine against two viral doses administrated by either central or peripheral routes. In parallel, seroconversion following pre-exposure vaccination was investigated. In this study, we demonstrated that the three investigated bat isolates were pathogenic, even at low dose, when inoculated by the central route but were not/less pathogenic when administrated peripherally. The Rabisin vaccine was capable of significantly cross-protecting mice inoculated intramuscularly with EBLV-1b and EBLV-2 and intracerebrally with BBLV. The level of rabies neutralizing antibodies induced by the Rabisin was quite high against the bat lyssaviruses, but with no significant differences between immunization with 1 and 5 IU/dose. The study emphasizes that the quality of rabies-inactivated vaccines for veterinary use is of utmost importance to optimize the cross-protection of pets against phylogroup I bat lyssaviruses occurring in Europe.

## 1. Introduction

After decades of rabies control programs, oral vaccination campaigns, and monitoring, most countries of Western, Central, and Northern Europe are now free of rabies transmitted by nonflying terrestrial mammals, such as foxes and raccoon dogs [1,2]. However, insectivorous bats still play an important role in the circulation of lyssaviruses in Europe [3], and rabies remains a concern in regards public health. Due to the increasing interest in bats and the pathogens they harbor [4], many countries have implemented or enforced epidemiological studies and national passive surveillance programs. Rabies in European bats were first reported in 1954 in Germany. For decades, lyssaviruses associated with European bats were only represented by a small number of species, such as the European bat lyssavirus 1 (EBLV-1) and the European bat lyssavirus 2 (EBLV-2). The majority of bat rabies cases are caused by EBLV-1, which is mainly associated with the serotine bat (*Eptesicus serotinus*) with 95% of the cases [5,6] and the meridional serotine (*Eptesicus isabellinus*) in Spain [7]. EBLV-1 circulates widely throughout Europe with two variants: variant “a” exhibiting an East–West distribution from Russia to the center of France with very little genetic variation, and variant “b” exhibiting a South–North distribution from Spain to Denmark and far more genetic diversity [6].

The distribution of EBLV-2, associated with Daubenton’s bats (*Myotis daubentonii*) and to a lesser extent in pond bats (*Myotis dasycneme*), is mainly limited to the United Kingdom (UK) and the Netherlands, but also to Finland, Germany, Switzerland, and, more recently, Norway [8]. The intensification of bat lyssavirus studies has led to the discovery of novel lyssaviruses in European bat populations. In 2010, Germany reported the isolation of a novel lyssavirus named Bokeloh bat lyssavirus (BBLV) in a Natterer bat (*Myotis nattereri*) [9]. Within 6 years, BBLV was further isolated twice in France in 2012 and 2013 [10], once in Poland in 2016 [11], and five more times in Germany. All BBLV cases were isolated from Natterer’s bats, with the exception of one case isolated in a common Pipistrelle bat (*Pipistrellus pipistrellus*) in Germany. The Lleida bat lyssavirus (LLBV) is another example of novel variants evidenced in bats. The LLBV was first reported in Spain during the summer of 2011 [12] in a common bent-winged bat (*Miniopterus schreibersii*) and is considered now as one of the most genetically divergent Lyssavirus spp. A second case of LLBV reported six years later in France, in a city 750 km from the initial Spanish case, raised questions about the virus’ distribution among Europe’s bat population [13]. Even more recently, a tentative novel member of the genus Lyssavirus, called Kotalahti bat lyssavirus, was detected in a Brandt’s bat (*Myotis brandtii*) in Finland [14], where only EBLV-2 cases were reported so far in Daubenton’s bat population. The phylogenetic analyses demonstrated that the Kotalahti bat lyssavirus virus was closely related to Khujand virus, Aravan virus, BBLV, and EBLV-2.

From 2010 to 2018, 330 bat rabies cases were reported in Europe (source: Rabies Bulletin Europe) i.e., an average of 36 cases reported annually, mainly by Germany, The Netherlands, Poland, and France. The transmission of European bat lyssaviruses to other animal species, including humans, is, fortunately, rare. For the time being, spillover infections to wildlife and domestic animals have only been reported for EBLV-1 in cats in France [15], stone marten in Germany [16], and sheep in Denmark [17]. In Europe, EBLV-1 and EBLV-2 caused four human deaths following bat exposures in Ukraine, Russia, Finland, and Scotland. None of these exposed people were vaccinated against rabies [18], nor received any postexposure treatment.

Rabies is preventable by vaccination. Current commercial rabies vaccines for veterinary and human use are mainly prepared from the original strain isolated by Pasteur in 1885 and all its derivates such as Pasteur virus (PV), Pitmann–Moore (PM), and challenge virus standard (CVS). These vaccines, and more especially HDCV (human diploid cell vaccine) and PVRV (purified vero rabies vaccine) vaccines for human use, have been shown to induce partial to complete protection (20% to 100% depending on the challenge route) against members of phylogroup I lyssaviruses such as EBLV-1, EBLV-2, or BBLV [19,20,21], but few/no protection against phylogroup II and III lyssaviruses [22]. Despite the fact that rabies inactivated veterinary vaccines are also prepared from RABV strains (PV more especially), few of them have been evaluated in cross-protection/cross-neutralization studies against bat lyssaviruses. Yet, because of the regular discovery of novel bat lyssaviruses in Europe, pet owners are wondering whether rabies veterinary vaccines, used to immunized dogs and cats, can protect their companion animals or not.

In the present study, we investigated the capacity of a widely used rabies inactivated veterinary vaccine (Rabisin, Boehringer-Ingelheim) to cross-protect mice experimentally infected with three different bat lyssaviruses occurring in Europe. We investigated two different dosages of vaccines, one of the two mimicking the minimum potency required for rabies veterinary vaccines (1 IU/dose or 1 IU/mL) and a high vaccine dosage mimicking a highly potent vaccine. Virus challenge has been carried out using central and peripheral routes at two different doses. In parallel, seroconversion following pre-exposure vaccination was investigated.

## 2. Materials and Methods

### 2.1. Virus

Three different bat lyssaviruses were used in this study including: one EBLV-1b isolated in 2000 on a serotine bat in France (belonging to the cluster B4, Genbank AY245838), one EBLV-2 isolated in 2002 on a *Myotis daubentonii* in United Kingdom (Genbank GU936871) [23], and one BBLV isolated in 2012 on a Natterer bat in France (belonging to the lineage A, Genbank KC169985) [24]. Initial bat lyssaviruses were isolated from bats and amplified on mice. The RABV isolate used in this study corresponds to a challenge virus standard 27 strain (CVS-27), adapted on a mouse model and commonly used for potency tests of rabies vaccines at the laboratory.

A comparison between the amino acid sequences of the glycoprotein from lyssaviruses used for challenge and from the PV vaccine strain indicated that this latter was 11.3%, 25.6%, 25.8%, and 29.4% divergent from CVS, BBLV, EBLV-2, and EBLV-1b respectively.

### 2.2. Vaccine

For evaluation of pre-exposure vaccination in mice, we used a commercial inactivated rabies vaccine for veterinary use (Rabisin Multi, Batch N° 15 RBNS 0591, Boehringer-Ingelheim). This batch was previously tested for potency (13 IU/mL) using a modification of the NIH test [25] as described in the monograph of the European Pharmacopoeia [26] and potency was estimated against the Biological Reference Preparation (BRP) batch N°5 [27] supplied by the European Directorate for the Quality of Medicines. From this estimated potency, the vaccine was diluted in sterile PBS to get two different doses used for the immunization step: a low dose adjusted to 1 IU/mL (mimicking the minimum potency required for rabies inactivated veterinary vaccines) and a higher dose of 5 IU/mL.

### 2.3. Animals

Animals used in this study consisted of Swiss OF-1 female mice (Charles River, France) weighing 13–15 g (about 3-weeks-old) on delivery. The characteristics of these mice (weight and strain) were similar to those required to conduct potency test of rabies inactivated vaccines for veterinary use (26). Mice were provided with food and water ad libitum and housed in an enriched environment in groups of 5 to 8 animals. All animals were monitored daily throughout the duration of the experimental procedures.

### 2.4. In Vivo Experiments

All in vivo experiments were conducted according to the regulation 2010/63/CE of the European Parliament and of the council of 22 September 2010 on the protection of animals used for scientific purposes [28], and as transposed into French law [29]. These experiments were covered by the Anses/ENVA/UPEC ethic committee, N°12-053 (13/11/2012).

### 2.5. Virus Titrations and Preparation of Challenge Doses

All virus strains tested in the present study were produced in mice. Virus production procedures were stopped when animals harbored symptoms suggestive of rabies stage 3/4 (convulsions, signs of paresis, or paralysis) [30] to collect a maximum amount of virus. For each virus, brains were excised from euthanized animals. Virus strains were prepared as brain supernatants and titrated in mice by the intracerebral (IC) and the intramuscular (IM) routes to determine the 50 MLD_50_ and the 2 MLD_50_ doses used for vaccine protection experiments.

### 2.6. Vaccine Protection Study

For the vaccine protection study, treatment groups comprised 8 mice. After 2 days of acclimatization, animals were vaccinated intraperitoneally with 0.5 mL of either a low dose (1 IU/mL) or high dose (5 IU/mL) of a Rabisin vaccine. At 2 weeks post-immunization (D14), animals were challenged intramuscularly in the masseter (i.m) or intracranially (i.c) with, respectively, 0.05 mL or 0.03 mL of either CVS-27, EBLV-1b, EBLV-2, or BBLV. Two different viral dosages were investigated: high dose (50 MLD_50_ per challenge dose) and low dose (2 MLD_50_ per challenge dose). In parallel, groups of 5 unvaccinated mice were challenged IM or IC with either the low dose or the high dose of each virus as controls and one group of 10 mice were vaccinated using a 5 IU/mL dose of Rabisin.

Animals were monitored daily for detection of clinical signs of rabies and scored as healthy, ill, or dead. To reduce the duration of animal suffering, humane endpoints were used instead of lethality. Animals were euthanized by CO_2_ intoxication when typical clinical signs of rabies, corresponding to stage 3 of the disease, were detected [30]. Experiments were terminated at day 35 (21 days after challenge).

Brains were collected post-mortem and assessed for the presence of rabies antigen using the fluorescent antibody test (FAT) [31].

Individual serum sample were taken by heart puncture after terminal anesthesia on animals that survived at the end of the experiment. After centrifugation of blood samples, sera were removed and stored at −20 °C until antibody assays.

For staff safety issues, blood sampling of animals showing rabies clinical signs were performed after euthanasia of mice, using filter papers soaked in the shredded heart [32].

To simply matters, the terms “mice died/succumbed from rabies” used in this article, should be seen as “mice that demonstrated rabies clinical signs and that were euthanized humanely”.

### 2.7. Virus Neutralizing Antibody (VNA) Induction in Vaccinated Unchallenged Mice

In parallel to the vaccine protection study, an additional group of 10 mice were vaccinated using a 5 IU/mL dose of Rabisin (batch 16 RBNS 0471) by the intraperitoneal route, to evaluate the possibility of a vaccine batch effect on the results. At 14 days post-vaccination, mice were anesthetized and bled by heart puncture. Sera were assessed for the presence of RABV neutralizing antibodies using the original FAVN test, as routinely done using the serological potency assay for the potency estimation of rabies inactivated vaccines for veterinary use [26].

### 2.8. Fluorescent Antibody Test

The detection of rabies antigens in the brain of mice was carried-out using the fluorescent antibody test (FAT), as described previously [31,33]. Basically, the whole brain of each mouse was used to impress slides. Impressions were dried at room temperature and fixed in cold acetone at –20 °C for 30 min. Slides were then air-dried and each impression was stained 30 min at 37 °C, in a high humidity chamber, with 50 µL of polyclonal fluorescein isothocyanate conjugate (lyophilised, adsorbed anti-rabies nucleocapsid conjugate, ref: 357-2112, Biorad, France). Slides were immersed and soaked in PBS for 5 min, rinsed with water, air-dried, and mounted by dropping a small amount of glycerolated buffer. Finally two trained laboratory technicians examined the slides under fluorescent microscope at 200× magnification.

### 2.9. Rabies Serological Assays

All serum samples were analyzed for rabies neutralizing antibodies using either the original FAVN test [34] (sera from mice infected with CVS-27) or a modified FAVNt (mFAVNt) for sera from mice infected with a bat lyssavirus.

Briefly, each sample (serum or eluate obtained from filter paper soaked with blood), as well as the positive (i.e., OIE positive reference serum) [35] and negative (ANSES collection) controls, were distributed in three or four consecutive wells, and then serially diluted. Due to the elution step, eluate samples had a starting dilution set at 1/27 whereas the serum samples had a starting dilution set at 1/3.

The challenge rabies virus (CVS-11, EBLV-1b, EBLV-2, or BBLV lyssaviruses) containing around a 50% tissue culture infective dose (TCID50) of 100 in 50 µL was then added to each well and was validated by back-titration. After 60 min of incubation, a volume of 50 µL of 4 × 10^5^ cells/mL suspension was added to each well and the microplates were incubated for 48 h at 36 ± 2 °C in a humidified incubator with 5% CO_2_. The microplates were stained by adding 50 µL of an appropriate dilution of a fluorescein isothiocyanate (FITC) anti-rabies monoclonal globulin (Fujirebio Diagnostics, Malvern, USA) to each well. Plates were qualitatively read according to an “all or nothing” method. The threshold of antibody detection was calculated by using the Spearman–Karber formula and set at 1.67 log D50 (logarithm of the dilution showing 50% inhibition of the positive wells) for the eluates (due to the starting dilution of 1/27) and at 0.84 for the sera. The titers of samples were expressed in international units per millilitre (IU/mL) by comparing results obtained with those of the positive reference standard. The threshold of positivity used was 0.5 IU/mL.

### 2.10. Statistical Analysis

For the pathogeny study, survival curves obtained for each group of mice challenged with the different rabies lyssaviruses were compared for statistical significance by the log rank test. For the cross-protection study, Fisher’s exact tests were used to analyze the survivorship differences between unvaccinated challenged groups and groups that were vaccinated and challenged. All analyses were conducted using the Graph Pad 6 software.

For the serological study, the one-sided limit test (Wilcoxon–Mann–Whitney’s exact test) and Mann–Whitney test were used to compare serological results for statistical significance. Calculations were performed with the Combistats Software version 5.0.

## 3. Results

### 3.1. Development of Clinical Disease in Unvaccinated Mice

**Figure 1:**
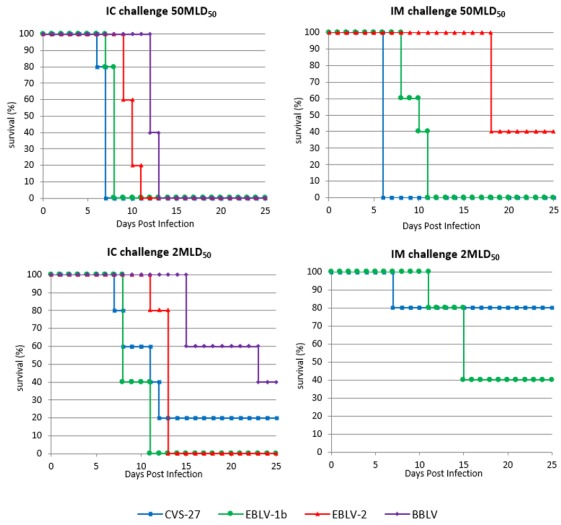
Pathogeny of challenge virus standard 27 strain (CVS-27), European bat lyssavirus 1 (EBLV-1b), European bat lyssavirus 2 (EBLV-2), and Bokeloh bat lyssavirus (BBLV) in unvaccinated Swiss OF-1 mice (*n* = 5 mice/group). Animals were challenged with either 50 MLD_50_ by the intracerebral (IC) route (**a**) and the intramuscular (IM) route (**b**), or 2 MLD_50_ by the IC route (**c**) and the IM route (**d**).

Whatever the virus used (CVS-27, EBLV-1b, EBLV-2, or BBLV) in control groups, all mice (100%) succumbed to rabies when challenged IC with 50 MLD_50_/30 µL. Death occurred between 6–7 days postinfection (p.i), 7–8 days p.i, 9–11 days p.i, and 12–13 days for CVS, EBLV-1b, EBLV-2, and BBLV, respectively (Figure 1a). When low dose (2 MLD_50_/30 µL) was administered IC, only EBLV-1b and EBLV-2 provided 100% fatalities in mice (Figure 1c). A survivorship of 20% and 40% was observed for mice infected with CVS and BBLV, respectively. As expected, at low challenge dose, the onset of symptoms and death was slightly increased whatever the virus used. Death occurred between 8–11 days p.i, 7–20 days p.i, 11–13 days p.i, and 15–23 days for EBLV-1b, CVS, EBLV-2, and BBLV, respectively.

Following peripheral inoculation, only CVS and EBLV-1b succeeded to cause 100% fatalities (Figure 1b) when administered IM at a high dose (50 MLD_50_/50 µL).

High dose IM of EBLV-2 led to 60% of mortality. Death occurred at 6 days p.i, between 8–11 days p.i, and at 18 days for CVS, EBLV-1b, and EBLV-2, respectively. Here again, the time to onset of symptoms and death was slightly increased at low dose (2 MLD_50_/50 µL). Death occurred at 7 days p.i and between 11–15 days p.i for CVS and EBLV-1b, respectively (Figure 1d). All mice challenged IM with low dose of EBLV-2 survived. It must be noted than none of the two doses of BBLV (50 or 2 MLD_50_/50 µL) caused fatalities in IM challenged mice even after one repetition of the experiment.

At high dose, survival curves were all significantly different between groups (*p* < 0.05, log-rank Mantel–Cox test) with a relative neurovirulence as follows: CVS > EBLV-1b > EBLV-2 > BBLV and CVS > EBLV-1b > EBLV-2 for the IC and the IM routes, respectively. At low dose, survival curves were not significantly different whatever the route of inoculation (central or peripheral), except for the EBLV-1b survival curve, which was significantly different from EBLV-2 (*p* = 0.008) and from BBLV (*p* = 0.0023) for the IC route.

All mice that showed clinical rabies signs in the control groups were diagnosed as positive for rabies using the FAT, whereas all mice that survived to the challenge (*n* = 12) were diagnosed negative.

### 3.2. Vaccination-Challenge Study

#### 3.2.1. Intracerebral Challenge

##### Protection

Vaccine administrated at the dose of 5 IU/mL resulted in a survival of 0%–71% in animals challenged by the IC route with 50 MLD_50_ of virus (Table 1). The level of protection provided by the high dose of Rabisin was better with CVS (71%) and then as follows: BBLV (50%) > EBLV-2 (13%) > EBLV-1b (0%). The efficacy of the high dose of vaccine was generally improved in animals challenged IC with a low dose of virus, except for EBLV-1b, where all immunized mice died from rabies. Protection reached 100% for CVS and BBLV and 38% for EBLV-2.

Vaccine diluted at the threshold of 1 IU/mL resulted in a survival of 0%–86% in animals challenged by the IC route with 50 MLD_50_ of virus. The level of protection followed the same pattern than the 5 IU/mL vaccine dose: CVS (86%) > BBLV (29%) > EBLV-2 = EBLV-1b (0%). As expected, the level of protection was globally equal or lower using a low dose of vaccine. On the contrary, although diluted at 1 IU/mL, the efficacy of the Rabisin vaccine was better in mice challenged with the low dose of virus: CVS = BBLV (100%) > EBLV-2 (25%) = EBLV-1b (13%).

The level of protection obtained against IC challenge was statistically significant for CVS (*p* < 0.05) whatever the vaccine and the challenge dose. For bat lyssaviruses, the level of protection was only statistically significant for BBLV administered at the dose of two MLD50.

The Rabisin vaccine totally failed to protect mice challenged with EBLV-1b, except one mouse that survived the low dose challenge.

##### Rabies Antigen Detection in Mice Brains

Brains of immunized/challenged mice were investigated for rabies antigen detection. FAT analyses revealed that 2 of the 52 mice (3.8%) that survived the virus challenge (one with a low dose of CVS-27 and one with a low dose of EBLV-2) were FAT-positive despite the absence of rabies clinical signs. These results were confirmed by RT-qPCR [36] (mean Ct = 28.9 ± 0.2 and 23.3 ± 0.05 respectively).

All vaccinated/challenged mice (*n* = 67) that succumbed to rabies were positive for the presence of rabies antigen in brain.

#### 3.2.2. Intramuscular Challenge

##### Protection

As BBLV did not cause any mortality when administered by the IM route, whatever the challenge dose (50 or 2 MLD50/dose), this route of administration was, therefore, not considered for the BBLV vaccination-challenge study. Similarly, the low dose (2 MLD50) of EBLV-2 failed to cause rabies in control group mice and was not investigated in the vaccination challenge-study.

Vaccines administrated at the dose of 5 IU/mL resulted in a survival of 43%–100% in animals challenged by the IM route with 50 MLD_50_ of virus (Table 1). The level of protection provided by the high dose of Rabisin was better with EBLV-2 (100%) and then followed by CVS (57%) and EBLV-1b (43%). The efficacy of the high vaccine dose was generally improved in animals challenged IM with a low dose of virus, with a protection reaching 100% for both CVS and EBLV-1b.

Vaccine diluted at the threshold of 1 IU/mL resulted in a survival of 38%–88% in animals challenged by the IM route with 50 MLD_50_ of virus with the following pattern: EBLV-2 (88%) > CVS = EBLV-1b (38%). As expected, the level of protection was globally equal or lower using a low dose of vaccine. On the contrary, when diluted at 1 IU/mL, the efficacy of the Rabisin vaccine was enhanced in mice challenged with the low dose of virus: CVS (100%) > EBLV-1b (75%).

The level of protection obtained against IM challenge was only statistically significant for low doses of EBLV-1b (*p* = 0.045) and for high doses of EBLV-2 (*p* = 0.035) in mice immunized with 5 IU/mL of vaccine.

##### Rabies Antigen Detection in Mice Brains

Diagnostic analyses indicated that brains of immunized/challenged mice were FAT positive and RT-qPCR positive (12.8 ± 0.0 < Ct values < 28.6 ± 0.29) for 6 of the 73 animals (8.2%) that survived to the virus challenge (three with a low dose of EBLV-1b and three with CVS-27). All mice that survived to IM challenge did not develop clinical signs of rabies.

All vaccinated/challenged mice (*n* = 20) that succumbed to rabies, or that were euthanized after onset of rabies signs, were positive for the presence of rabies antigen in brain.

#### 3.2.3. Virus Neutralizing Antibody Response in Mice

The mean VNA titers obtained for the two “vaccine control” groups of mice (Table S1), were respectively 41.8 IU/mL (range 23.9–54.8, *n* = 10, batch 15 RBNS 0591) and 48.6 IU/mL (range 3.46–95.27, *n* = 10, batch 16 RBNS 0471) and were not significantly different (*p* = 0.309).

All vaccinated mice that survived to the challenge (100%, *n* = 125) developed a positive virus neutralizing antibody (VNA) response at the end of the experiment. Mice challenged with CVS-27 (IC and IM) had a mean VNA titer of 75.4 IU/mL (range 13.8–287, *n* = 22) and 43 IU/mL (range 1.99–165, *n* = 25) when immunized with five (high) and one (low) IU/dose vaccines respectively, with this difference being significant (*p* = 0.007) (Table 2a).

When challenged with EBLV-1b, EBLV-2, or BBLV, mice also developed a higher mean VNA response when immunized with the high dose of Rabisin with mean titers equal to 48.2 IU/mL (*n* = 10), 21.4 IU/mL (*n* = 11), and 9.2 IU/mL (*n* = 20) for EBLV-1b, BBLV, and EBLV-2, respectively. However, whatever the bat lyssavirus, the VNA response was not significantly different from the response observed in mice immunized with the low dose of Rabisin (0.115 < *p* < 0.232).

It should be noted that the mean VNA response in mice challenged with CVS-27 and immunized with a high vaccine dose was always significantly higher (data not shown) than the responses obtained in mice challenged with EBLV-1b, EBLV-2, and BBLV and immunized at the same dose (*p*-values ranging from 0 to 0.007). Similarly, this VNA response was significantly higher (*p* = 0.017) than the mean responses observed for the “vaccine control groups”. For mice immunized with 1 IU/dose vaccine, the difference between CVS-27 and bat lyssaviruses was significantly different for EBLV-2 and BBLV only (*p* = 0.000).

**Figure 2:**
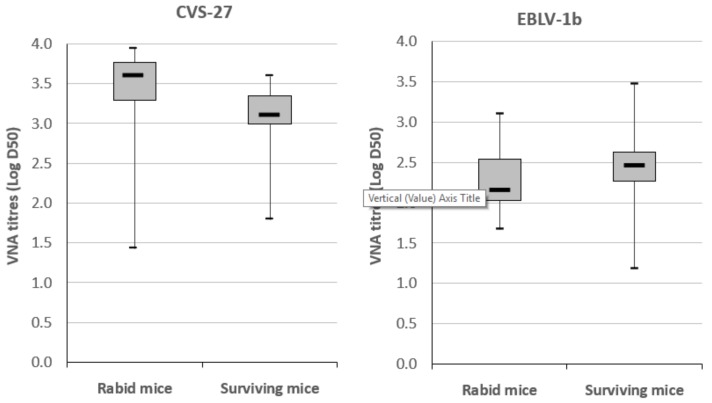

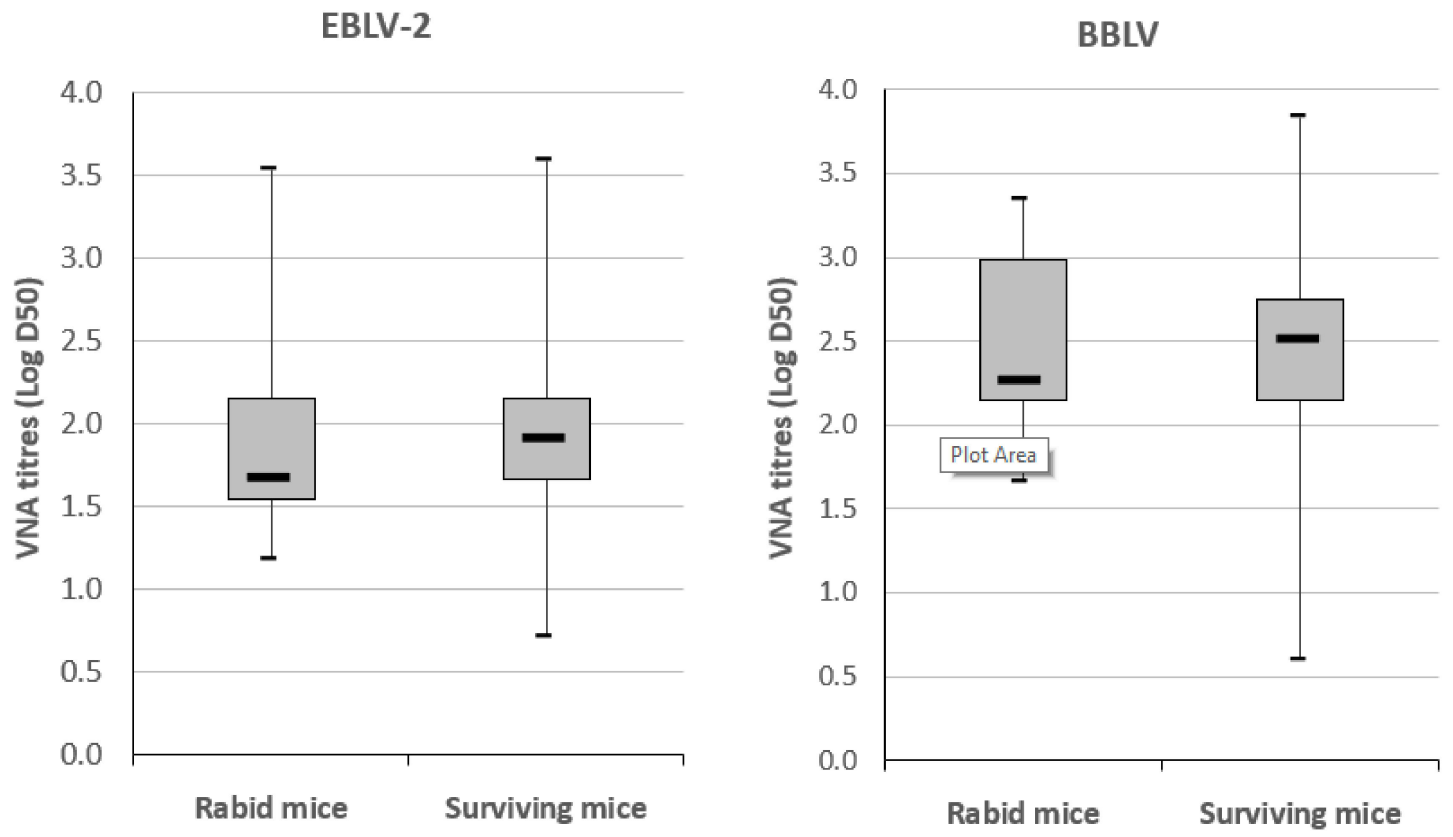
Comparison of VNA titers (log D50) in immunized mice that survived or succumbed to lyssavirus challenge (CVS-27, EBLV-1b, EBLV-2, or BBLV). Boxes represent the first and third quartiles and the line within the box represents the median value. Whiskers above and below the box show the locations of the minimum and maximum values.

Vaccinated mice that were euthanized after the onset of clinical signs of rabies were also investigated for VNA. In total, 83.5% of mice (*n* = 71/85) had detectable levels of VNA with no significant differences between immunization at 5 IU/dose or 1 IU/dose (Table 2b), whatever the virus used for challenge. When compared to VNA responses obtained in surviving mice at the end of the experiment (Figure 2), no significant differences were reported whatever the virus used for the challenge (median test, Mann–Whitney, 0.053 < *p*-value < 0.82), but the distributions of values had a larger spread in surviving mice that were challenged with bat lyssaviruses.

## 4. Discussion

Currently, sixteen lyssavirus species are recognized by the International Committee on the Taxonomy of Viruses and segregated into three phylogroups [37]. RABV is part of the phylogroup 1 and is the main causative virus for human, and for the time being, the only virus used to produce rabies vaccines for human and veterinary use. Different strains of fixed RABV are generally used for vaccine production. Among others, the most common strains are the Pasteur Virus (PV) strain adapted to Vero cells, Pitman–Moore (PM) strain adapted to human diploid and Vero cells, CVS adapted to BHK-21 cells, Flury LEP (low egg passage), or HEP (high egg passage) Flury adapted to chick embryo cells [38]. Currently, these vaccines may not be totally efficient to protect against lyssaviruses that do not belong to phylogroup I [22].

Cross protection of classical rabies (RABV) vaccines against other phylogroup I lyssaviruses has been investigated in numerous studies. It has been demonstrated that rabies vaccines for human use (mainly HDCV and PVRV) were capable of providing partial protection against bat lyssaviruses such as Duvenhage, EBLV-1, EBLV-2, ABLV, or BBLV [19,20,21,39]. In these studies, cross protection of rabies inactivated vaccines for veterinary use has rarely been investigated. Moreover, vaccine doses, challenge virus doses, and time between immunization and challenge are quite heterogeneous, some of them being more or less close to the recommendations of monographs for determining the potency of rabies vaccines.

In this study, we investigated the capacity of a widely used rabies inactivated veterinary vaccines for pets (Rabisin, Boehringer-Ingelheim) to cross-protect mice experimentally infected with three different bat lyssaviruses occurring in Europe. We attempted to use a protocol that mimicked as much as possible of the pre-exposure/challenge model on mice used in Europe for batch release of veterinary rabies vaccines [40] by predicting their efficacy on target species. We investigated two different doses of vaccines, one of the two mimicking the minimum potency required for rabies veterinary vaccine (1 IU/dose or 1 IU/mL) [26] and a high vaccine dosage representing a highly potent vaccine. Virus challenge has been carried out at two different doses: one representing the dose commonly recommended for vaccine potency test on mice (50 MLD_50_) [26] and a less severe dose (2 MLD_50_) that has been demonstrated to induce clinical signs of rabies.

Data from this study have shown that all investigated virus strains were highly pathogenic when administered at a high dose through the IC route and resulted in 100% mortality in mice with mean survival times ranked as follows: shortest CVS > EBLV-1b > EBLV-2 > BBLV longest. Mean survival times were globally delayed at a lower IC challenge dose and for the IM route. Whereas it was pathogenic when inoculated IC, the French BBLV strain did not induce clinical signs when inoculated IM, which contrasts the results obtained in a previous study with two German BBLV isolates [19]. In this latter study, despite their close genetic relatedness with the French isolate (sequence analysis of the N and G genes showed more than 98% identity between the French KC169985 isolate and the German JF311903 isolate) [10], these German isolates led to 80%–100% mortality when administered IM at low and high doses in a BALB/C mouse strain. This difference of pathogenicity remains puzzling. Similarly, the low IM dose of EBLV-2 failed to cause rabies in mice.

Following pre-exposure rabies vaccination of mice, a significant partial/total protection was observed for mice challenged IC with CVS-27, whatever the vaccine dosage used for the immunization. For bat lyssaviruses, pre-exposure rabies vaccinations lead to heterogeneous levels of protection according to the strain used for challenge, the route of administration, and the challenge dose. In our conditions, the Rabisin vaccine globally failed to protect mice challenged IC with EBLV-1b (survival of 0%–13%) but succeeded in offering a partial protection when the peripheral route was used. For EBLV-2, a lack of efficacy was also demonstrated for high dose IC challenge, but partial protection seemed to be induced by the vaccine in mice inoculated IC with a low challenge dose, with the protection being better using the peripheral challenge route. Cross-protection against BBLV was only investigated for the IC route since the lyssavirus failed to induce rabies intramuscularly. Although it was partial (29%–50%) for the severe challenge dose, a total and significant protection must be noted at a low challenge dose whatever the vaccine dosage.

Data obtained using the mFAVN test revealed that mice sera were capable of cross-neutralizing the investigated bat lyssaviruses EBLV-1b, EBLV-2, and BBLV. All immunized mice that survived to the viral challenge (IC and IM) developed positive VNA responses (≥0.5 IU/mL), with the highest values exceeding 100 IU/mL for CVS-27 and EBLV-1b. Cross-VNA levels are thought to be a result of antigenic distance between the vaccine strain and the challenge virus: the more homologous the vaccine strain is to the challenge virus, the higher is the protective efficacy. Here, CVS-27 showed the closest genetic relationship to the PV vaccine strain, followed by BBLV, EBLV-2, and EBLV-1b. Nevertheless, there was no correlation evidenced between the genetic distance from PV (% amino acids identity of the glycoprotein G) and the neutralizing capacity of the anti-G PV sera. Nevertheless, CVS-27 VNA levels were significantly higher than the ones obtained for the three bat lyssaviruses.

The figures obtained in the present study seem to be higher than those obtained in previous studies [19,20,21] with HDCV/PVRV vaccines, but the comparison is quite hampered since vaccine dosages, number of immunizations, duration of experiments, etc. are quite different from one experiment to another. The adjuvant contained in veterinary vaccines such as Rabisin, a compound that is not found in human rabies vaccines, plays a key role in the magnitude and breadth of specific immune responses to antigens. It improves the efficacy of vaccines by enhancing neutralizing antibodies or the duration of the protective response [41]. In a recent study [21], Nokireki et al. demonstrated that the VNA responses obtained with a single dose of Rabisin in EBLV-2 challenged mice was as efficient as a double injection of a HDCV vaccine, a commercial human vaccine based on a Pitman–Moore fixed rabies virus (closely related to the PV strain used in Rabisin). High titers observed here may confirm that the efficacy of rabies vaccines is rather due to this difference in vaccine formulation than to the vaccine strain itself [22].

Our data demonstrated that the Rabisin vaccine was capable of eliciting positive VNA responses in mice challenged with either EBLV-1b, EBLV-2, or BBLV. The level of detectable VNA afforded by a 1 IU dose in surviving mice, although inferior, was not significantly lower than the VNA levels obtained with a 5 IU immunization. Both vaccine doses were unable to offer significant protection of mice inoculated intracerebrally with EBLV-1b and EBLV-2. However, the intracerebral route is a severe form of challenge that does not reflect the situation of a natural infection, with the severity of this route being accentuated when using high doses of virus such as 50 MLD_50_. Nevertheless, the Rabisin vaccine, even diluted at a dose of 1 IU was able to provide significant protection against BBLV in this severe condition. Cross-protection evaluation through the peripheral route is a more realistic approach to mimic natural infection. In our conditions, the Rabisin vaccine was able to provide a total and significant protection of mice infected IM with either a high dose of EBLV-2 or a low dose of EBLV-1b. When diluted to 1 IU, the level of protection afforded by the vaccine slightly decreased in groups inoculated with bat lyssaviruses. Even if VNA responses are hardly correlated to the protection observed in experimental studies, this clearly suggests that the selection of the vaccine dilution is a critical issue in cross-protection experiments.

From the data obtained in this study, we can conclude that inactivated rabies vaccines, with potency respecting the minimum threshold of 1 IU/dose, are capable of eliciting some cross-protection against EBLV-1b, EBLV-2, and BBLV occurring in Europe, the level of protection being dependent upon the bat lyssaviruses. Rabies veterinary vaccines with a high potency are more likely to afford a better protection in contrast to those with a potency around the threshold. This is of particular importance in a context where ineffective rabies vaccines (for either human or veterinary use) are regularly reported [42].

## Figures and Tables

**Table 1:** Cross protection of rabies inactivated veterinary vaccine in mice challenged with 4 different rabies virus species by central/peripheral route at two different dosages.

		Survivorship after Challenge			
Virus		IC Route n (%)		IM Route n (%)	
		50MLD_50_	2MLD_50_	50MLD_50_	2MLD_50_
CVS	Control group	0/5 (0)	1/5 (20)	0/5 (0)	4/5 (80)
	Vaccine 5 UI	5/7 * (71)	5/5 * (100)	4/7 * (57)	8/8 (100)
	*p*-value	0.028	0.048	0.081	0.385
	Vaccine 1 UI	6/7 * (86)	8/8 (100)	3/8 (38)	8/8 (100)
	*p*-value	0.015	0.007	0.231	0.385
EBLV-1b	Control group	0/5 (0)	0/5 (0)	0/5 (0)	2/5 (40)
	Vaccine 5 UI	0/7 * (0)	0/7 * (0)	3/7 * (43)	7/7 * (100)
	*p*-value	1	1	0.205	0.045
	Vaccine 1 UI	0/7 * (0)	1/8 (13)	3/8 (38)	6/8 (75)
	*p*-value	1	1	0.231	0.293
EBLV-2	Control group	0/5 (0)	0/5 (0)	2/5 (40)	nc
	Vaccine 5 UI	1/8 (13)	3/8 (38)	8/8 (100)	nc
	*p*-value	1	0.231	0.035	/
	Vaccine 1 UI	0/8 (0)	2/8 (25)	7/8 (88)	nc
	*p*-value	1	0.487	0.216	/
BBLV	Control group	0/5 (0)	2/5 (40)	nc	nc
	Vaccine 5 UI	4/8 (50)	7/7 * (100)	nc	nc
	*p*-value	0.105	0.046	/	/
	Vaccine 1 UI	2/7 * (29)	8/8 (100)	nc	nc
	*p*-value	0.47	0.036	/	/

* Group in which ≥1 mouse died from inoculation traumatisms, but not from rabies. These animals were not taken into account. *p*-values calculated from the Fisher’s exact test for the ratio of vaccinated mice that survived to the viral challenge and compared with the ratio of mice that survived to the challenge in respective control group (significant *p*-values are underlined); nc: experiment has not been conducted

**Table:** (**a**) Surviving mice

	Mean VNA (IU/mL)		
Challenge Virus	Immunization 5 IU/Dose	Immunization 1 IU/Dose	*p*-Value
CVS-27	75.4 (13.8–287)	43.0 (2–165)	0.007
EBLV-1b	48.2 (6.7–244)	21.8 (1.3–35.4)	0.232
EBLV-2	9.2 (0.5–33)	7.2 (0.1–29.4)	0.133
BBLV	21.4 (1.3–81.1)	11.2 (0.1–35.4)	0.115

**Table:** (**b**) Euthanized mice

	Mean VNA (IU/mL)		
Challenge Virus	Immunization 5 IU/Dose	Immunization 1 IU/Dose	*p*-Value
CVS-27	63.2 (0.9–125.6)	167.2 (41.6–287)	nc
EBLV-1b	15.9 (3.8–45.6)	16 (6.6–34.6)	0.422
EBLV-2	18.9 (2.7–97.5)	4.4 (1.2–10.9)	0.056
BBLV	41.2 (1.7–81.1)	11.5 (5.1–35.4)	0.151

*p*-values derived using the one-sided limit test (Wilcoxon–Mann–Whitney’s exact test) for the VNA titers of mice immunized with 5 IU/dose vs. VNA titers of mice immunized with 1 IU/dose. Values in brackets indicate the minimum and the maximum VNA titer observed in the group. nc: not calculated.

## Supplementary Materials

The following are available online at https://www.mdpi.com/1999-4915/11/10/936/s1, Table S1: RVNA titres (in IU/ml) assayed with the FAVN test in control group mice vaccinated with a RABISIN batch.
