# Supplementary material for: Cross-Protection of Inactivated Rabies Vaccines for Veterinary Use against Bat Lyssaviruses Occurring in Europe
Source: Viruses. 2019 Oct 11;11(10):936. doi: 10.3390/v11100936 (PMC6832384; doi:10.3390/v11100936)
Supplement: Supplementary file 1 [file viruses-11-00936-s001.pdf]

Table S1

RVNA titres (in IU/ml) assayed with the FAVN test in control group mice vaccinated with a RABISIN batch

Control group vaccinated with batch 16RBNS0471	
**Mouse**	**RVNA titer (UI/ml)**
mouse 1	41,59
mouse 2	31,55
mouse 3	72,27
mouse 4	41,59
mouse 5	3,46
mouse 6	72,27
mouse 7	72,27
mouse 8	41,59
mouse 9	95,27
mouse 10	13,77

Control group vaccinated with batch 15RBNS0591	
**Mouse**	**RVNA titer (UI/ml)**
mouse 1	41,59
mouse 2	54,82
mouse 3	31,55
mouse 4	41,59
mouse 5	31,55
mouse 6	23,93
mouse 7	54,82
mouse 8	41,59
mouse 9	54,82
mouse 10	41,59
